# Bilateral macular hole secondary to remote lightning strike

**DOI:** 10.4103/0301-4738.57156

**Published:** 2009

**Authors:** Krishna A Rao, Lavanya G Rao, Ajay N Kamath, Vikram Jain

**Affiliations:** Department Of Ophthalmology, Kasturba Medical College, Manipal, India

**Keywords:** Lightning, macular hole, posterior subcapsular cataract

## Abstract

We report a case of a 16-year-old girl, who was struck by lightning, and experienced blurred vision in the right eye (RE) immediately following the episode. She reported for ophthalmic evaluation two months later. Examination revealed relative afferent pupillary defect in the RE. Posterior subcapsular cataract was noted in both eyes. Fundus examination revealed macular holes and multiple areas of RPE hyperpigmentation in the periphery in both eyes. Fundus fluorescein angiography showed increased choroidal transmission with early fluorescence and late fading in the foveal region and retinal pigment epithelium (RPE) stippling in the periphery in both eyes. This is the first case report of such nature in India to the best of our knowledge.

More than half of all lightning victims suffer from some form of ophthalmic injury, most commonly involving the cornea.[1] Anterior segment injuries include thermal keratopathy, uveitis, hyphema, anterior and posterior subcapsular cataract, and dislocated lens.[2] Posterior segment injuries include vitreous hemorrhage, retinal edema, retinal hemorrhage, retinal detachment, cystoid macular edema, chorioretinal rupture, lightning maculopathy, macular hole, central retinal vein occlusion and central retinal artery occlusion.[2–4] Neurological injuries include thermal papillitis, optic neuropathy, loss of pupillary reflex, anisocoria, Horner's syndrome, multiple cranial nerve palsies and nystagmus.[2,4]

We report a case of lightning-induced ocular injury involving anterior and posterior segments and also causing neurological injury.

## Case Report

**Figure 1:**
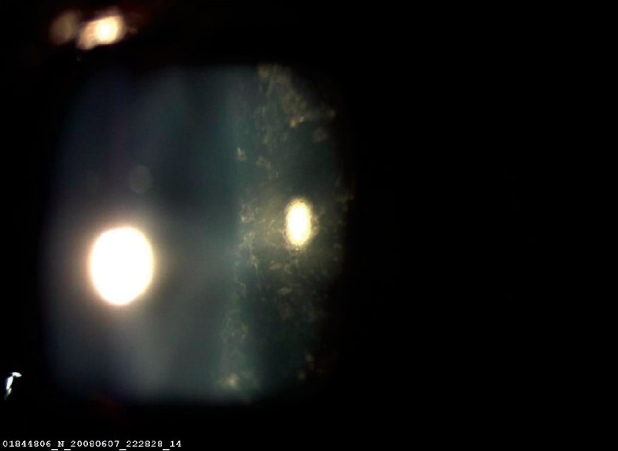
Right eye posterior subcapsular cataract

**Figure 2:**
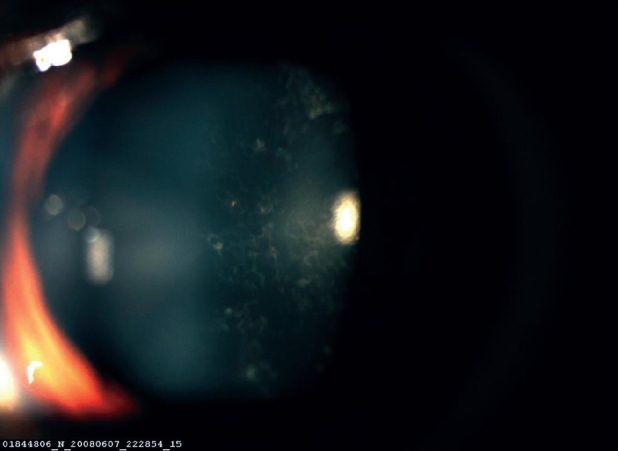
Left eye posterior subcapsular cataract

**Figure 3:**
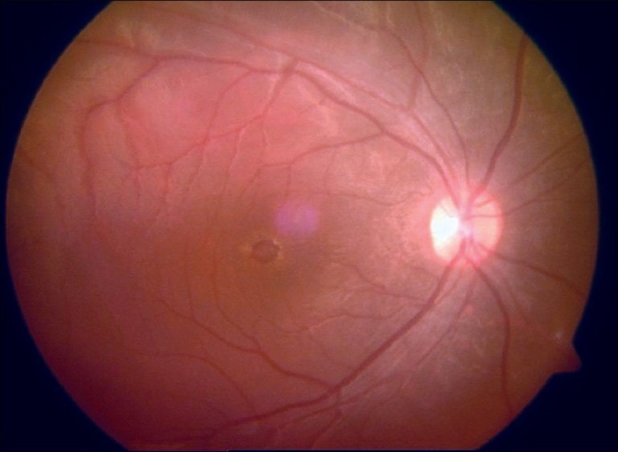
Color fundus photograph of right eye showing macular hole

**Figure 4:**
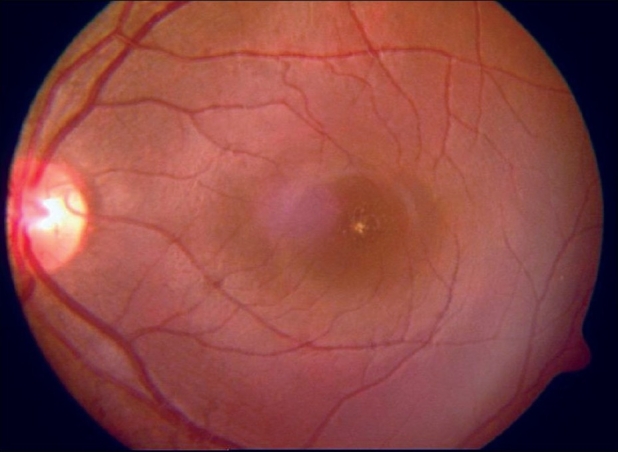
Color fundus photograph of left eye showing macular hole

**Figure 5:**
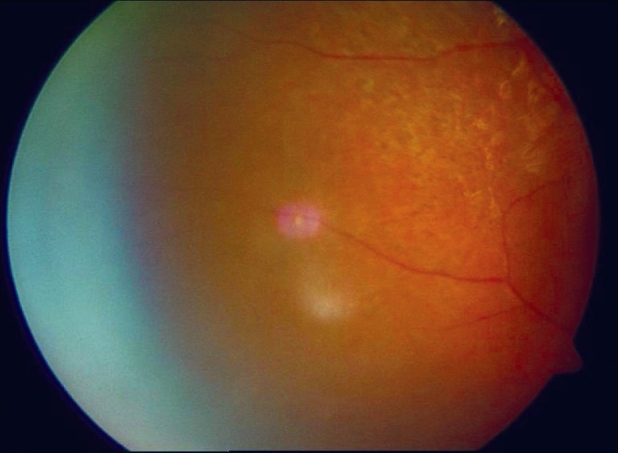
Color fundus picture of the periphery showing RPE hyperpigmentation

**Figure 6:**
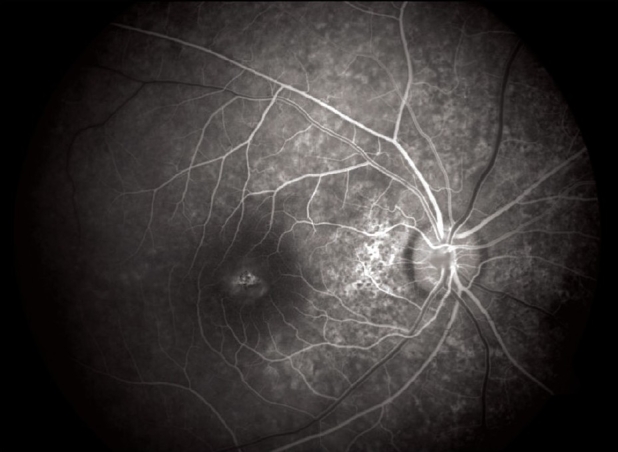
FFA picture of right eye showing foveal window defect

**Figure 7:**
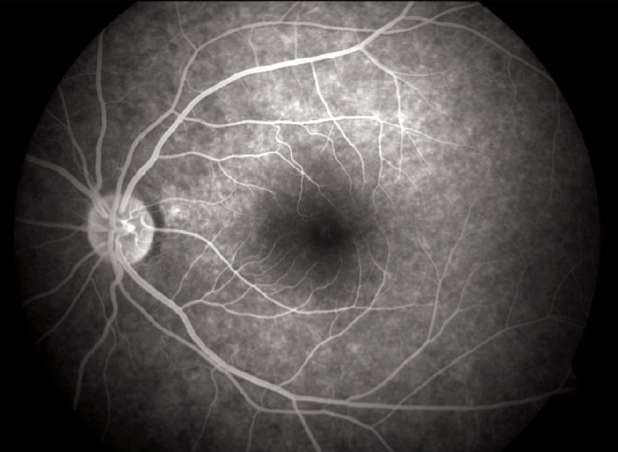
FFA picture of left eye showing foveal window defect

**Figure 8:**
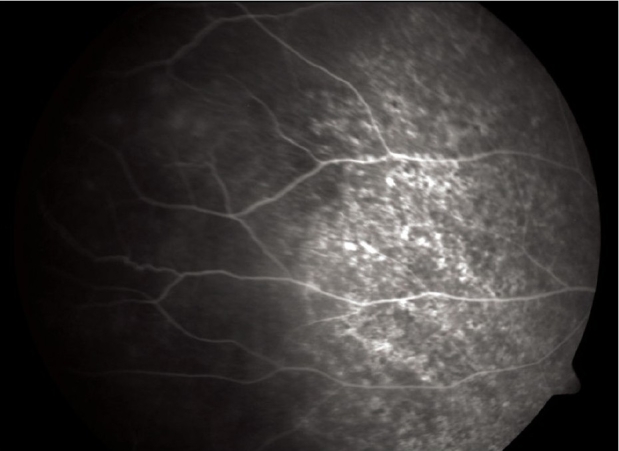
FFA picture of the periphery showing RPE stippling

A 16-year-old girl presented to us with sudden painless diminution of vision in the right eye (RE) following a lightning strike while sleeping in the house on the ground at night two months ago (April 2008). Her brother-in-law, who was sleeping approximately six feet away died immediately but this girl survived and experienced immediate decreased vision in the RE and loss of hearing in the right ear. However, she reported for ophthalmic evaluation two months later in June 2008. On examination, her best-corrected visual acuity was 20/80, N18 in RE and 20/30, N10 in left eye (LE). Intraocular pressure was 12 and 10 mm Hg respectively. Anterior segment examination revealed RE relative afferent pupillary defect (RAPD). Posterior subcapsular cataract was noted in both eyes [Figs. 1 and 2]. Her fundus examination revealed bilateral macular holes [Figs. 3 and 4] and multiple areas of retinal pigment epithelium (RPE) hyperpigmentation in the periphery in both eyes [Fig. 5]. Posterior vitreous detachment or operculum was not found. Fundus fluorescein angiography showed increased choroidal transmission with early fluorescence and late fading in the foveal region and RPE stippling in the periphery in both eyes [Figs. 6–8]. General physical examination showed stable pulse, blood pressure and renal function. Our patient did not sustain any skin or eyelid burns. On her last visit in July 2008, she was found to have RE anterior uveitis and has been treated with topical steroids and cycloplegics.

## Discussion

Lee *et al*.[4] reported four routes by which lightning reaches its victims and causes injuries:

1. Direct strike: when the major current flows directly through the victim and is facilitated by metal objects.
2. Splash: where lightning strikes an object first and then arcs through the path of least resistance.
3. Contact: when lightning strikes an object the victim is in contact with such as being electrocuted while talking over the phone or in the bathtub by current flowing through wires or pipes.
4. Ground current: the bolt strikes the ground and travels along the surface towards the victim.

Our patient probably sustained the injury by the fourth mechanism mentioned - ground current passing initially through the right side of the body indicated by loss of hearing in right ear, decreased vision RE (20/80) more than LE (20/30), denser posterior subcapsular cataract in RE, and presence of RAPD in RE. Lightning might have caused optic nerve damage leading to degeneration causing RAPD in RE, however, no such damage has occurred in the LE. Papillitis is a known manifestation of lightning injury.[2,4] Our patient might have had optic neuritis, which may have resolved by the time she reported to us.

Lightning involves a transfer of electric charge. Also lightning contact is instantaneous taking less time (exposure time usually lasts only 1 to 100 milliseconds) to cause injury.[5] Tissue is destroyed by both heat and electrolysis. The high resistance offered by non-nervous tissue accounts for the thermal effects of electrical injuries, which result in immediate coagulation of the proteins of the cells.[6] Electrical cataract from lightning is bilateral in most of the cases. The closer the distance of the contact area to the eye, the higher the likelihood of cataract formation. In bilateral cases, cataract formation will start in the eye on the affected side and the time difference between cataract occurrences in the two eyes can be as long as 1–10 months. The proposed mechanisms[7,8] of cataract formation due to lightning are the following:

- Decreased permeability of lens capsule.
- Protein coagulating effect of electrical current.
- Nutritional disturbance of lens due to iritis.
- Mechanical damage to the lens fibers.

Morphologically, lightning cataract is quite characteristic. Both anterior and posterior parts of the lens can be affected due to lightning. Clinically, the cataract formation may vary considerably. Regression of the whole opacity or part of the opacity may be seen in some cases.[7,8] In our case there was no evidence of anterior subcapsular cataract. Only posterior subcapsular cataract was found in both eyes, RE more than LE.

The high temperature generated can cause carbonification of the skin and the underlying tissue. When the current traverses the skin, energy from the current is converted into heat, producing coagulation and necrosis of the striated muscles and blood vessels through which it passes.[9] However, our patient did not sustain any skin or eyelid burns.

The macula is very sensitive to thermal damage because of the high content of melanin granules of its RPE, which constitute the main obstacle to the current flow.[3–4] Electrical current could damage RPE by electrolysis. Also, melanin acts as a resistor to electric current serving to heat these tissues, producing thermal denaturation of the outer retina and RPE. Lightning can also emit a cylindrical shockwave which can mechanically injure the pigment epithelium. Localized inflammation in response to lightning injury could contribute to retinal pigment epithelial dysfunction. Intraretinal edema could result from decreased transport of fluid out of retina or development of retinal vascular incompetence.[3] Macular edema simulating Berlin's edema seen early after lightning strike may be replaced by lesions described as a ‘cyst’, ‘macular hole’ or ‘solar maculopathy’.[3–4] Initial macular holes due to lightning injury may undergo spontaneous closure subsequently as reported by Lee *et al*.[4] It is important to differentiate between macular cystic changes and full-thickness macular hole due to lightning, because maculopathy with cystic change may spontaneously resolve but for full-thickness macular hole surgical intervention is sometimes recommended.[10] Handa *et al*.[3] state that lack of posterior vitreous detachment and operculum support the diagnosis of lightning maculopathy as opposed to full-thickness macular hole. Optical coherence tomography (OCT) is an important diagnostic tool for the differentiation between the two entities. Visual prognosis in patients with lightning-induced ocular injury will depend upon the extent of involvement of ocular structures and in the absence of anterior segment manifestation, irreversible retinal damage as well as optic nerve damage are the major determinant factors. The unpredictability of electric flow through the human body must have resulted in the posterior subcapsular cataracts, macular holes and RPE stippling in our patient.
